# Association of dietary pattern with biochemical blood profiles and bodyweight among adults with Type 2 diabetes mellitus in Tehran, Iran

**DOI:** 10.1186/s40200-015-0155-0

**Published:** 2015-04-15

**Authors:** Nasrin Darani Zad, Rokiah Mohd Yusof, Haleh Esmaili, Rosita Jamaluddin, Fariba Mohseni

**Affiliations:** Department of Nutrition and Dietetics, Faculty of Medicine and Health Sciences, Universiti Putra Malaysia, 43400 UPM, Serdang Selangor, Malaysia; Endocrinology and Metabolism Research Center, Tehran University of Medical Science, Tehran, Iran

**Keywords:** Dietary pattern, Factor analysis, Diabetes mellitus, Food frequency questionnaire

## Abstract

**Background:**

This study was conducted to identify dietary patterns and evaluated their association with biochemical blood profiles and body weight among adults with type 2 diabetes mellitus.

**Methods:**

This was a cross sectional study conducted among 400 patients with type 2 diabetes mellitus in Tehran from March to August 2013. Biochemical blood profiles, socio-demographic, lifestyle, anthropometric measurements, and dietary data were obtained. Dietary data from food frequency questionnaire were used to derive dietary patterns. Factor analysis was conducted to ascertain the dietary patterns, and analysis of covariance was fitted to assess the relation between blood profiles, body weight and adherence to dietary patterns.

**Results:**

Three dietary patterns by factor analysis were identified, Vegetable & Poultry, Western and Semi-healthy. After control for potential confounders, body mass index (b = −0/03, p < 0.05) were negatively associated with vegetable and poultry dietary pattern. Conversely, total cholesterol (b = 0.004, p < 0.01) and fasting blood glucose (b = 0.014, p < 0.05) were positively associated with western dietary pattern. A dietary pattern labeled as semi-healthy pattern was found to be positively related to HDL-cholesterol (b = 0.006 p < 0.01). Associations between semi-healthy pattern, LDL-cholesterol (b = −0.120 p < 0.05) and waist circumference (b = −0.020, p < 0.05) were negative.

**Conclusion:**

Adherence to Vegetable & Poultry dietary pattern was favorably related to body weight, semi healthy related to lower LDL and higher HDL cholesterol whereas western related to higher fasting blood glucose and total cholesterol. Further studies are necessary to confirm the benefits of the dietary patterns for diabetes.

## Background

Type 2 diabetes mellitus (T2DM) is a well-known disease in both developed and developing countries. It is reported that most patients with type 2 diabetes could have dyslipidemia at varying degrees, characterized by increased levels of triglyceride (TG) and LDL Cholesterol and decreased HDL Cholesterol when this characteristic lipid profile is seen in type 2 diabetes, it is referred to as diabetic dyslipidemia and confers a risk of *cardiovascular disease* (CVD). Diet plays an important role in diabetes management [1]. Some studies have described the associations between diabetes mellitus and diet, in terms of single nutrients [2-4] or food groups [5]. However, the single nutrients or foods alone can explain only a part of the effects of diet on diabetes. Therefore, the terms of dietary pattern has been recommended as an approach used to investigate diet-disease relations. The dietary pattern approach is a powerful means for summarizing nutrient and food intake to depict the whole diet [6,7]. The aim of the present study was identified the dietary patterns and investigated dietary patterns in relation to biochemical blood profiles and body weight among adults with type 2 diabetes mellitus.

## Materials and methods

This cross-sectional study was performed in the frame work of a project approved by Faculty of Medicine and Health Science of the Universiti Putra Malaysia and Special Medical Center in Tehran, Iran. In this project 400 samples of male and female adults with T2DM in Tehran, Iran. Subjects were invited to participate and written informed consent was obtained from each subject. A stratified random sampling method was used for subject selection within the age range of 40–60 years old and data was collected from one of the biggest diabetic centers in the capital city of Iran. Subjects with a history of CVD, cancer, or any chronic disease, except diabetes mellitus, were excluded because of possible disease-related changes in diet. Before performing the main study, a pilot study was performed on 30 subjects who were not included in the main study. The aim of the pilot study was to minimize errors and biases in collecting data. The sociodemographic characteristics used in this study were (age, sex, marital status, education, occupation and income) and lifestyle characteristics (smoking and physical activity), some other related factors (duration of DM, diabetes treatments, hypertension, family history of DM). Subjects provided information on age (years), sex (male, female), marital status (single, married, widow, or divorced), education levels (uneducated, primary school, secondary school, high school, or university degree), occupation (housewives, the government, the private section, retired, or business owners), monthly income (low, average, or high), smoking status and physical activity, duration of DM (year), diabetes treatments (oral agents, insulin or oral agents plus insulin), hypertension (yes, no), family history of DM (yes, no). For each subjects, we determined their physical activity level in weekly metabolic equivalent hours as measured using the International Physical Activity Questionnaire (IPAQ short form). It was validated in Persian language by Ataei et al. [8]. These data were extracted from the general questionnaire through face-to-face interviews carried out by a trained dietitian. To measure height to the nearest 0.1 cm, subjects were asked to take off their shoes and stand with their shoulders in a normal position; while subjects’ weight was measured to the nearest 0.1 kilogram without shoes and wearing minimal clothes. Waist circumference (WC) was measured at the narrowest level, and weight (kg) was divided by the square of height (m) to calculate the body mass index (BMI). The average of three measurements was used as the result and presented in this study. After a 12-h overnight fast, we drew blood sample for biochemical assessment. Levels of total cholesterol (T cholesterol), triglyceride (TG), HDL cholesterol, LDL cholesterol, hemoglobin A1c (HbA1c), and fasting blood glucose (FBG) were analyzed. All the tests were done by commercial kits (all from Pars Azmoon, Iran) using an auto-analyzer system (Selectra E, Vitalab, the Netherlands). HbA1c was determined using colorimetric method after an initial chromatographic separation (Bio Systems, Spain).

The analysis of data used the Statistical Package of Social Science (SPSS for windows, Version 16). To measure the dietary intake of the subjects, a semi-quantitative food frequency questionnaire (FFQ) [9], including 105 food items was used. Their FFQ was modified by removing some items that were rarely consumed (<5%) by our sample. The FFQ included a list of foods (with standard serving sizes) commonly consumed by Iranians it was based on the frequency of consumption of each item from a list of foods, for a period of time ranging from a month to a year, and it is a simple and inexpensive dietary assessment method which is the most frequently used tools in epidemiological studies nowadays [10]. Subjects had a choice of reporting their intake either in terms of reference portion size or in grams. A reference portion, representing one standard serving expressed in household measures, was defined for each food item. Common household measures, such as measuring cups, spoons and portion size photos, were used for better estimation of the real portion consumed. Data were collected by trained dietitians during a structured interview, using a questionnaire. The subjects were asked to answer how often they had consumed each of the food items throughout the preceding year to the study. Five categories, from daily (1 or more), 3–5 times per week, 1–2 times per week, 1–2 times per month and never, were used to record the frequency for the consumption of each food item. Participants who left >50 items blank on the food-frequency questionnaire or participants who reported a total daily energy intake outside the mean range 3 standard deviations of energy intake were excluded. The reported frequency of each food item and beverage was then converted to frequency intake per day. The portion sizes of foods used by subjects were changed to grams. Then, the grams of each food item were multiplied by the frequency intake per day to compute the grams of each food intake per day. For the purpose of determining dietary patterns, food items were grouped into twenty-three food groups, on the basis of similarities in ingredients, nutrient profile and/or culinary usage. Food items having a unique composition (eggs and tea) were classified individually (Table 1). The total consumption for each group was determined by summing up the daily portion intake of each item in this group. Dietary patterns were identified using exploratory principal component factor analysis (PCFA) from the twenty-three food groups. PCFA is a data-driven method that identifies foods that are frequently consumed together by pooling food items on the basis of the degree to which the amounts eaten are correlated together [11]. Before running the factor analysis procedure, the correlation matrix among the twenty three food groups was visually and statistically examined to justify undertaking factor analysis. The Bartlett test of sphericity was significant at P < 0.05, and the Kaiser–Meyer–Olkin (KMO) test showed a score of 0.62, indicating that the correlation among the variables was sufficiently strong for a factor analysis. Orthogonal transformation was applied to generate major dietary patterns. With the criteria of >1.5 Eigenvalues, further inspection of the scree plot showed a clear break after the third component. The derived dietary patterns were labeled on the basis of food groups having a rotated factor loading of >0.3 [12]. Factor scores were calculated by summing the intake of each food group weighted by factor loading [13] and each individual received a factor score for each dietary pattern. The relation between blood profiles, WC and BMI and adherence to the dietary patterns was assessed using analysis of covariance. Data were adjusted for sociodemographic factors (age, sex, education, household income, occupation, marital status), lifestyle factors (smoking and physical activity), duration of diabetes mellitus, treatment of diabetes mellitus, family history of diabetes, hypertension, energy intake (kilojoules per day), and body mass index.

**Table 1:** **Food groupings used in the dietary pattern analysis**

**Food group**	**Food items**
**Refined grains**	White Iranian breads (lavash, taftoon) noodle, pasta, rice, baguettes
**Legumes**	Beans, peas, split peas, broad beans, lentils, soy
**Eggs**	Eggs
**Fruit**	Pears, apricots, cherries, apples, grapes, bananas, cantaloupe, watermelon, oranges, melon, kiwi, strawberries, peaches, lime, fresh figs, dates tangerine, plums, persimmons, pomegranates, lemons, sour cherry
**Nuts**	Peanuts, pistachios, hazelnuts, sesame, Sunflower seeds, walnuts, chick-pea (roasted),
**Sweets and desserts**	Chocolates, simple cakes (simple cake made of egg, flour, sugar,), biscuit, halva (an iranian sweet made of oil, flour and sugar), cube sugar, candies, gaz (an Iranian confectionery made of glucose, nuts, and tamarisk),fruit conserved with glucose as dessert
	Jam, jelly, honey, dry pastries, creamed pastries,
**Hydrogenated fats**	Hydrogenated fats, animal fats
**Fish**	Canned tuna fish, other fish, shrimps
**High-fat dairy product**	Cream, dried whey, cheese, ice cream, pizza cheese
**Tea**	Tea
**Processed meat**	Sausages, cold cut
**Pizza**	Pizza
**French fries**	French fries, chips
**Potatoes**	Cooked and barbecued Potatoes
**Olive**	Olives and olive oils
**Green leafy vegetables**	Lettuce and spinach
**Yellow vegetables**	Carrots and carrot juice
**Tomatoes**	Tomatoes
**Other vegetables**	Cucumber, semi-healthy vegetables, eggplant, green beans, green pepper, squash, mushrooms, cooked and raw onions
**Poultry**	Chicken with or without skin, quail
**Red meats (low fat)**	Beef, hamburger, lam

## Results

Factor 1 was characterized by higher intake of fruit, fish, poultry, low fat dairy, green leafy vegetable, tomato, yellow vegetables, other vegetable and olive oil was labeled as vegetables and poultry dietary pattern. Factor 2 was labeled as western dietary pattern which was heavily loaded on legumes, sweets, egg, fish, high fat dairy product, French fries, potatoes, yellow vegetables, and sweets. Factor 3 was characterized by high consumptions of refined grain, fruits, nuts, tea, red meat and olive oil and by a low consumption of whole grain such that it was labeled as the ‘semi-healthy’ dietary pattern (Table 2). Together these three dietary patterns accounted for 31.19% of the variances in the original dietary intake. The first pattern as vegetable and poultry pattern was the most dominant food pattern in the sample and explained 12.55% of the variance in dietary intake, whereas each of the 2 other Factors explained 10.24 and 8.40 respectively. General characteristics of subject by dietary pattern are shown in Table 3. In this study, female patients and those with primary education (b = 0.30, P < 0.05) presented higher adherence to the ‘vegetable and poultry’ pattern, compared to males and those with a university degree. Patients with hypertension were more likely to follow the ‘vegetable and poultry’ pattern. A profile of being a male (b = 0.28, p < 0.05), and non-smokers (b = 0.59 p < 0.05), as compared to female, ex-smokers, and those with less physical activity (b = −0.81, p < 0.001) were associated with the ‘western’ pattern. Patients who had a higher income and those with family history of diabetes were more likely to have the ‘semi-healthy’ pattern. Multivariate analysis of biochemical blood profiles and body weight associated with adherence to the three dietary patterns are presented in Table 4. We defined BMI (b = −0/03, p < 0.05) was negatively associated with vegetable and poultry dietary pattern. Conversely, total cholesterol (b = 0.004, p < 0.01) and FBG (b = 0.014, p < 0.05) were positively associated with western dietary pattern. HDL cholesterol (b = 0.006 p < 0.01) was found to be positively related and, WC (b = −0.020, p < 0.05) and LDL cholesterol (b = −0.120 p < 0.05) were negatively associated with semi-healthy dietary pattern.

**Table 2:** **Factor loading matrix for dietary patterns identified by principal component analysis**

**Food groups**	**Factors and factor loadings**		
	**Vegetable and poultry dietary pattern**	**Western dietary pattern**	**Semi-healthy dietary pattern**
Refined grain	−	−	.347
Legumes	−	.539	−
Eggs	−	.451	−
Fruit	.384	.262	.328
Nuts	−	−	.393
Sweets	−	.580	−
Hydrogenated fat	−	−	−
Fish	.348	.444	−
High fat dairy	−	.336	−
Tea	−	−	.492
Processed meat	−	−	−
French fries	−	.524	−
Potatoes	−	.497	−
Poultry	.532	−	−
Low fat dairy	.376	−	−
Whole grains	−	−	-.455
Red meats (low fat)	−	.246	.429
Green, leafy vegetables	.613	−	−
	−	−	−
Pizza	−	.491	−
Tomato	.618	-.273	−
Yellow vegetable	.394	.372	−
Other vegetables	.707	−	−
Olive	.326	.222	.323

Values ≤0.30 were excluded for simplicity.

**Table 3:** **Factors associated with adherence to major dietary patterns among subjects with type 2 diabetes mellitus**

**Vegetable and poultry Western Semi-healthy**						
	**b**	**95% CI**	**b**	**95% CI**	**b**	**95% CI**
Age	0.004	-.007 to .01	.004	-.01 to .01	-.003	-.01 to .01
Sex						
Male	−0.33*	−0.57 to -.08	.280*	0.0 to .56	-.28	-.67 to .09
Female	0 (ref)					
Marital status						
Married	−0.42	−1.11 to .27	.058	-.74 to .86	.07	-.72 to .86
Single	−0.37	−1.237 to .49	-.565	−1.57 to .44	.29	-.70 to 1.28
Widow	−0.36	−1.12 to .39	-.078	-.96 to .80	-.09	-.96 to .78
Divorce Education level	0 (ref)					
Uneducated	-.35	-.68 to .083	-.65*	−1.21 to -.08	-.18	-.74 to .37
Primary	.30*	.08 to.51	-.16	-.63 to .20	-.08	-.54 to .38
Secondary	-.31	-.65 to .02	-.26	-.73 to .20	.17	-.29 to .63
High school	-.29	-.59 to -.002	.13	-.24 to .52	-.07	-.45 to .30
University degree Occupation	0 (ref)					
government section	-.68	−1.44 to .89	-.27	−1.44 to .89	.01	−1.14 to 1.17
Household	-.07	-.54 to .45	-.04	-.54 to .45	-.001	-.49 to .49
Private section	-.33	-.49 to .57	.03	-.49 to .57	.16	-.36 to .69
Retired	-.17	-.74 to .25	-.24	-.74 to .25	.20	-.28 to .70
Business owners	0 (ref)					
Monthly income						
<500$	.26	-.27 to .79	.24	-.36 to .86	-.70*	−1.31 to -.09
500-1000$	.08	-.40 to .57	.21	-.35 to .78	-.66	−1.22 to -.10
1000$<	0 (ref)					
Family history of Diabetes mellitus	.05	-.16 to .26	-.13	-.38 to .11	.34*	.09 to .59
Hypertension Diabetes treatment	.20*	.15 to .38	-.15	-.37 to .69	-.12	-.33 to .09
oral agent	-.05	-.32 to .21	.37*	0.05 to .68	.07	-.23 to .38
Insulin	-.23	-.62 to .14	.07	-.37 to .52	.17	-.26 to .61
Both Smoking	0 (ref)					
Current	−30	−1.05 to .43	.13	-.79 to .99	.41	-.43 to 1.26
Non	-.47	-.99 to .03	.59*	.005 to 1.19	.04	-.54 to .63
Ex- smoker	0 (ref)					
Total physical activity (MET)	0.002	-.010 to 0.01	−0.81*	−1.8 to -.44	-.004	-.016 to 0.007
Duration of Diabetes	0.006	-.008 to .02	-.004	-.20 to .01	.003	-.01 to .01

b, regression coefficient (a positive coefficient implies greater adherence to the pattern); CI, confidence interval; MET, metabolic equivalent task, ref = reference  Data were adjusted for sociodemographic (age, sex, education, household income, occupation, marital status), lifestyle (smoking, physical activity), duration of diabetes mellitus, treatment of diabetes mellitus,, family history of diabetes and hypertension, *P < 0.05.

**Table 4:** **Association between Dietary Pattern with Biochemical Blood Profiles and weight status**

**Vegetable and poultry Western Semi-healthy**									
	**b**	**95% CI**		**b**	**95% CI**		**b**	**95% CI**	
Hemoglobin A1c	-.007	-.013	.000	-.0003	-.099	.099	.071	-.028	.171
Fastingblood glucose	-.002	-.005	.000	.014*	.024	.003	-.003	-.005	.000
Triglyceride	.002	-.000	.003	-.001	-.003	.000	.0008.	-.001	.002
Total cholesterol	-.004	-.009	.002	.004*	.000	.010	.004	-.001	.009
LDL cholesterol	.003	-.003	.009	-.001	-.007	.005	-.120*	-.669	-.085
HDL cholesterol	.003	-.008	.014	.001	-.001	.004	.006*	.005	.017
Body mass index	-.034*	-.067	-.002	0.01	0.002	0.028	0.08	_0.11	0.28
Waist circumference	0.061	-.318	.196	0.03	0.004	0.069	-.020*	-.034	-.007

b, regression coefficient (a positive coefficient implies greater adherence to the pattern); CI, confidence interval;  Data were adjusted for sociodemographic (age, sex, education, household income, occupation, marital status), lifestyle (smoking, physical activity), duration of diabetes mellitus, treatment of diabetes mellitus, family history of diabetes, hypertension, energy intake (kilojoules per day), and body mass index. *P < 0.05.

## Discussion

Three dietary patterns were identified, named ‘vegetable and poultry’, ‘western’, and ‘semi-healthy’. The ‘vegetable and poultry’ pattern was similar to ‘Healthy’ and ‘Mediterranean’ patterns in other studies [9,14]. The vegetable and poultry dietary pattern was found to be related to lower BMI. Other studies have reported [15,16] found the healthy dietary pattern which was high in fruit, high-fiber cereal, reduced-fat dairy products, whole grains, low in red, processed meats, and fast food, showed smaller gains in BMI. Factor 2 labeled as western dietary pattern was similar to western dietary pattern mentioned in various studies [13,9]. In this study, western dietary pattern was associated with, higher FBG and Total Cholesterol. Some studies demonstrated that western dietary pattern was positively associated with FBG [17,18]. Van Dam et al. [18] identified a similar pattern labeled as traditional (high in red meat and potatoes, low in fruit and low-fat dairy) that was positively associated with plasma glucose. Lower consumption of fruits and higher consumption of starch foods were significantly associated with an increased risk of high FBG [19]. In our study, we have high loading of fish on western dietary pattern, and we observed positive association between blood glucose with fish intake. It is surprising that high intake of fish may have influence on the increase of blood glucose. There are some possible reasons for explaining this finding as frying method, the amount and type of oil used [20] and greater consumption of salt [21]. Total Cholesterol was positively associated with western dietary pattern. The meat and western dietary patterns were associated with high Total Cholesterol levels [22]. In addition, high daily consumption of eggs, a rich source of dietary cholesterol increases Total Cholesterol levels [23,24]. In a prospective study of men; it was suggested that they are good sources of dietary fiber with a positive effect on concentration of Total Cholesterol [25], and contributes to improved glycaemic control but the results of our study showed that consumption of legumes had direct correlation to a dietary pattern with the cholesterol and blood glucose increase, as most of the legumes intake was attributable to intake lentils, peas or beans stew together with meat. Factor 3 labeled as semi-healthy dietary pattern, The ‘semi-healthy ‘pattern, included with high loadings on fruits, nuts, tea, whole grains, olive oil, red meat and refined grains. In the present study, the pattern that included healthy foods also included refined grains and red meat, which are not considered healthy; for this reason, this pattern was designated as semi-healthy. In our study semi-healthy dietary pattern was positively associated with HDL cholesterol. It was negatively associated with WC and LDL cholesterol. Some studies showed that dietary pattern characterized by a healthy balance diet (with a frequent intake of salad vegetables, fruits, fish, pasta, rice and low intake of fried foods, sausages, fried fish and potatoes) were inversely correlated with central obesity [13,26] and positively correlated with HDL cholesterol [27]. In this study, major monounsaturated fatty acid (MUFA) in semi-healthy dietary pattern was oleic acid, found in olive oil and nuts. Different studies support the beneficial effects of MUFAs on lipid profiles mainly, decreased LDL cholesterol [28]. Furthermore, inclusion of tea in a diet moderately low in fat reduces LDL cholesterol by significant amounts [29].

This study contains several strengths. It was population-based and used a validated food frequency questionnaire. In addition, to our knowledge, this is the first to identify the dietary pattern of diabetic patients in the Middle East. This study also has several limitations. We performed a cross-sectional design in which hinders any casual inferences difficult. There are several methods for the assessment of food and nutrient consumption and energy intake. All methods have advantages and limitations and the choice depends on the purpose for which the information is intended. In this study, measurement errors inherent in the use of FFQs for dietary assessment include possible under-reporting or over-reporting of general food intake, selective under-reporting or over-reporting of the intakes of certain foods, or both. Since, there were no suitable Iranian foods tables; the USDA National Nutrient Database tables for Standard Reference were used in this study.

## Conclusion

In conclusion, the emergence of dietary pattern analysis is a complementary and alternative approach for examining the association between the chronic diseases and diet. This study identified three major dietary patterns. We determined dietary patterns were associated with biochemical blood profiles and body weight among Iranian with T2DM. It was observed that patients that have adhered to a semi-healthy dietary pattern had a higher HDL cholesterol and lower LDL cholesterol and whereas a negative relation between WC, BMI and the Vegetable & Poultry dietary pattern. Adherence to a western-type dietary pattern is associated with higher T-cholesterol and FBG concentrations in diabetic patients. The planning of public health nutrition policy and designing of preventive nutrition intervention to tackle diabetes complication, which is Iranian’s leading cause of mortality, should follow the understanding of dietary exposure. Diet, physical activity status and socio- demographic factors should be taken into consideration when designing based diabetes prevention programs; in addition, more studies are needed to work on dietary patterns among diabetic patients and confirm their benefits for them.

## Abbreviations

T2DM: Type 2 diabetes mellitus
ANCOVA: Analysis of covariance
FFQ: Food frequency questionnaire
FBG: Fasting blood glucose
HbA1c: Hemoglobin A1C
HDL: High density lipoprotein
KMO: Kaiser-Meyer-Olkin
LDL: Low density lipoprotein
MET: Metabolic equivalent
PCA: Principal component analysis
SPSS: Statistical package for the social sciences
TG: Triglyceride
TCholestrol: Total cholesterol

## References

[1] American association of diabetes (2008). Nutrition recommendations and interventions for diabetes (position statement). Diabetes Care.

[2] Jenkins DJ, Kendall CW, Marchie AL, Jenkins AL, Augustin LS, Ludwig DS (2003). Type 2 diabetes and the vegetarian diet. Am J Clin Nutr.

[3] Shahar DR, Abel R, Elhayany A (2007). Does dairy calcium intake enhance weight loss among overweight diabetic patients?. Diabetes Care.

[4] Nor munirah MY, Siti Shafurah A, Norazmir MN, Hayati adilin MAM, Ajau D (2012). Roles of whole grains -based products in maintaining treatment targets among type 2 diabetes mellitus patients. Asian J Clin Nutr.

[5] Brand-miller J, Hayne S, Petocz P, Colagiuri S (2003). Low–glycemic index diets in the management of diabetes. Diabetes Care.

[6] Franz MJ, Bantle JP, Beebe CA, Brunzell JD, Chiasson LJ, Garg A (2002). Evidence-based nutrition principles and recommendations for the treatment and prevention of diabetes and related complications. Diabetes Care.

[7] Lim JH, Lee YS, Chang HC, Moon MK, Song Y (2011). Association between dietary patterns and blood lipid profiles in korean adults with type 2 diabetes. J Korean Med Sci.

[8] Ataei F, Fazeli M, Tanhaei A, Shabani A, Ashtari L, Baghaki A (2004). Making Persian version of international physical activity questionnaire. MedSport.

[9] Esmaillzadeh A, Kimiagar M, Mehrabi Y, Azadbakht L, Hu FB, Willett WC (2007). Dietary patterns, insulin resistance, and prevalence of the metabolic syndrome in women. Am J Clin Nutr.

[10] Lima F, Latorre M, Costa M, Fisberg RM (2008). Diet and cancer in northeast brazil: evaluation of eating habits and food group consumption in relation to breast cancer. Cad Saude Publica.

[11] Naja F, Nasreddine L, Itani L, Chamieh MC, Adra N, Sibai AM (2011). *l*. Dietary patterns and their association with obesity and sociodemographic factors in a national sample of lebanese adults. Public Health Nutr.

[12] Mccann SE, Marshall JR, Brasure JR, Graham S, Freudenheim JL (2001). Analysis of patterns of food intake in nutritional epidemiology: food classification in principal components analysis and the subsequent impact on estimates for endometrial cancer. Public Health Nutr.

[13] Rezazadeh A, Rashidkhani B, Omidvar N (2010). Association of major dietary patterns with socioeconomic and lifestyle factors of adult women living in tehran, iran. Nutrition.

[14] Panagiotakos DB, Tzima N, Pitsavos C, Chrysohoou C, Zampelas A, Toussoulis D (2007). The association between adherence to the mediterranean diet and fasting indices of glucose homoeostasis: the attica study. Am J Clin Nutr.

[15] Newby P, Muller D, Hallfrisch J, Andres R, Tucker KL (2004). Food patterns measured by factor analysis and anthropometric changes in adults. Am J Clin Nutr.

[16] Okubo H, Sasaki S, Murakami K, Kim M, Takahashi Y, Hosoi Y (2007). Three major dietary patterns are all independently related to the risk of obesity among 3760 japanese women aged 18–20 years. Int J Obes (Lond).

[17] Wirfalt E, Hedblad B, Gullberg B, Mattisson L, Andren C, Rosander U (2001). Food patterns and components of the metabolic syndrome in men and women: a cross-sectional study within the malmo diet and cancer cohort. Am J Epidemiol.

[18] RM V d, Grievink L, Ocké MC, Feskens EJM (2003). Patterns of food consumption and risk factors for cardiovascular disease in the general dutch population. Am J Clin Nutr.

[19] Paek KW, Chun KH, Lee SJ (2011). A factor of fasting blood glucose and dietary patterns in korean adults using data from the 2007, 2008 and 2009 korea national health and nutrition examination survey. J Prev Med Public Health.

[20] Patel PS, Sharp SJ, Luben RN, Khaw KT, Bingham SA, Wareham NJ (2009). Association between type of dietary fish and seafood intake and the risk of incident type 2 diabetes: the european prospective investigation of cancer (epic)-norfolk cohort study. Diabetes Care.

[21] Nanri A, Mizoue T, Yoshida D, Takahashi R, Takayanagi R (2008). Dietary patterns and a1c in japanese men and women. Diabetes Care.

[22] Sadakane A, Tsutsumi A, Gotoh T, Ishikawa S, Ojima T, Kario K (2008). Dietary patterns and levels of blood pressure and serum lipidsin a japanese population. J Epidemiol.

[23] Weggemans RM, Zock PL, Katan MB (2001). Dietary cholesterol from eggs increases the ratio of total cholesterol to high-density lipoprotein cholesterol in humans: a meta-analysis. Am J Clin Nutr.

[24] Djouss’e L, Gaziano JM, Buring JE, Lee LM (2009). Egg consumption and risk of type 2 diabetes in men and women. Diabetes Care.

[25] Bazzano LA, Thompson AM, Tees MT, Nguyen CH, Winham DM (2011). Non-soy legume consumption lowers cholesterol levels: a meta-analysis of randomized controlled trials. Nutr Metab Cardiovasc Dis.

[26] Hamer M, Mishra GD (2010). Dietary patterns and cardiovascular risk markers in the uk low income diet and nutrition survey. Nutr Metab Cardiovasc Dis.

[27] de Williams PA, Whichelow MJ, Cox BD, Day NE, Wareham NJ (2000). A cross-sectional study of dietary patterns with glucose intolerance and other features of the metabolic syndrome. Br J Nutr.

[28] Cernea S, Hancu N, Raz I (2003). Diet and coronary heart disease in diabetes. Acta Diabetol.

[29] Davies MJ, Judd JT, Baer DJ, Clevidence BA, Paul D, Edwards AJ (2003). Black tea consumption reduces total and ldl cholesterol in mildly hypercholesterolemic adults. J Nutr.

